# Extrahepatic Iron Loading Associates With the Propensity to Develop Advanced Hepatic Fibrosis in Hemochromatosis

**DOI:** 10.1016/j.gastha.2024.01.011

**Published:** 2024-01-24

**Authors:** John K. Olynyk, Timothy G. St. Pierre, James Chen, David M. Frazer, Louise E. Ramm, Grant A. Ramm

**Affiliations:** 1 Curtin Medical School, Curtin University, Bentley, Western Australia, Australia; 2 Department of Gastroenterology, Fiona Stanley Hospital, Murdoch, Western Australia, Australia; 3 School of Physics, Mathematics, and Computing, University of Western Australia, Crawley, Western Australia, Australia; 4 Molecular Nutrition Laboratory, QIMR Berghofer Medical Research Institute, Herston, Queensland, Australia; 5 School of Biomedical Sciences, Queensland University of Technology, Gardens Point, Queensland, Australia; 6 School of Biomedical Sciences, The University of Queensland, St Lucia, Queensland, Australia; 7 Faculty of Medicine, The University of Queensland, Herston, Queensland, Australia

**Keywords:** HFE, Liver Fibrosis, Hemochromatosis, Iron

## Abstract

**Background and Aims:**

Hemostatic iron regulator-hemochromatosis can result in progressive iron-loading and advanced hepatic fibrosis in some individuals. We studied total body and hepatic iron loading to determine whether the distribution of iron-loading influences the risk of advanced fibrosis.

**Methods:**

One hundred thirty-eight men and 66 women with hemochromatosis who underwent liver biopsy for staging of hepatic fibrosis had evaluation of hepatic iron concentration (HIC), hepatic iron index (HIC/age), total body iron stores (mobilizable iron), and mobilizable iron/HIC ratio (a marker of total body iron relative to hepatic iron). The potential impact of liver volume on mobilizable iron stores was assessed using magnetic resonance imaging in a separate cohort of 19 newly diagnosed individuals with hemochromatosis.

**Results:**

Of 204 biopsied subjects, 41 had advanced fibrosis and exhibited 60% greater accumulation of mobilizable iron relative to HIC (mean 0.070 ± 0.008 g Fe/[μmol Fe/g]) compared with 163 subjects with low-grade fibrosis (mean 0.044 ± 0.002 g Fe/[μmol Fe/g], *P* < .0001). Linear regression modeling confirmed a discrete advanced hepatic fibrosis phenotype associated with greater mobilizable iron stores relative to HIC. The ratios of the upper to lower 95% limits of the distributions of liver volumes and the mobilizable iron/HIC ratios were 2.7 (95% confidence interval 2.3–3.0) and 9.7 (95% confidence interval 8.0–11.7), respectively, indicating that the distribution of liver volumes is not sufficiently wide to explain the variability in mobilizable iron/HIC ratios, suggesting that significant extrahepatic iron loading is present in those with advanced hepatic fibrosis.

**Conclusion:**

Advanced hepatic fibrosis develops in hemostatic iron regulator-hemochromatosis individuals who also have excessive extrahepatic mobilizable iron stores.

Hemostatic iron regulator (HFE)-hemochromatosis is a common disorder of iron metabolism caused by a homozygous mutation (p.C282Y) of the *HFE* gene, which can result in significant clinical manifestations and iron overload in up to 40% of men and 12% of women.^1^^,^^2^ Just over half of these individuals may progress to develop advanced hepatic fibrosis or cirrhosis (Scheuer liver fibrosis stages F3 or F4,^1^, ^2^, ^3^, ^4^, ^5^ respectively, now termed ‘advanced hepatic fibrosis’), increasing the risk of hepatocellular carcinoma, which is a principal contributor to morbidity and mortality in this condition.^6^ The iron overload observed in hemochromatosis results from suppression of hepcidin production, the key liver-derived negative regulator of iron absorption from the gastrointestinal tract and iron release from body iron stores. The homozygous p.C282Y mutation in HFE results in absence of the protein and a loss of the ability to regulate hepcidin synthesis, causing inappropriately low serum hepcidin levels, providing the connection between the genetic mutation and pathogenesis of iron overload in hemochromatosis.^1^

The risk of developing advanced hepatic fibrosis in individuals with hemochromatosis is thought to be related to the duration and extent of iron overload, although this is highly variable.^7^, ^8^, ^9^ Bassett et al introduced the concept of a threshold of hepatic iron concentration (HIC), above which the likelihood of advanced hepatic fibrosis or cirrhosis became much more likely.^10^ Thereafter, advancing age, higher serum ferritin level, greater duration of exposure to elevated HIC, and greater level of mobilizable iron stores emerged as additional risk factors.^1^^,^^7^^,^^11^, ^12^, ^13^, ^14^ However, what has proven difficult to reconcile is the high variability of HIC to accurately predict the level of mobilizable iron stores,^7^^,^^8^ especially in the setting of advanced hepatic fibrosis. Early studies of hemochromatosis, prior to discovery of the underlying genetic mutation, demonstrated bone marrow iron overload in up to 60% of subjects with significant iron overload and cirrhosis.^15^ Valberg et al showed that there is dissociation in the rate of iron accumulation between the bone marrow and liver in clinical hemochromatosis, with excess iron being deposited predominantly and preferentially in the liver until the later stages of disease, when bone marrow deposition occurs.^16^ Thus, it is possible that quantitation of iron deposition in the liver, via measurement of HIC, may not accurately reflect the degree of iron loading occurring elsewhere in the body, such as the bone marrow, accounting for the variability previously reported.^7^^,^^8^ To examine this possibility, we studied total body and hepatic iron loading to determine whether the distribution of iron loading between the liver and bone marrow explains this variability and is associated with the risk of advanced hepatic fibrosis.

## Methods

### Patients

We studied 138 men and 66 women with C282Y homozygous HFE-hemochromatosis recruited at the Royal Brisbane and Women’s Hospital and QIMR Berghofer Medical Research Institute in Australia between 1983 and 2013 as part of routine standard of care for assessment of hemochromatosis.^7^ To be eligible for study inclusion, all subjects required complete baseline demographics, clinical information (presence or absence of arthritis or diabetes mellitus), total number of phlebotomies, alcohol consumption, serum biochemistry, liver biopsy histology, and HIC. HIC was measured by atomic absorption spectrophotometry on fresh liver biopsy specimens and expressed as ***μ***mol Fe/g dry weight (abbreviated to ***μ***mol Fe/g).^17^ The National Health and Medical Research Council of Australia definition for alcohol consumption (1 standard drink contains 10g of alcohol) was used to record alcohol consumption. All subjects were routinely recommended to undergo a liver biopsy as part of baseline assessment during the study period. Phlebotomy treatment was performed weekly on all subjects following liver biopsy to reach a serum ferritin level of 50 to 100μg/L. We calculated the hepatic iron index (HII, HIC/age), total body mobilizable iron stores (mobilizable iron = number of phlebotomies x 250 mg to reduce ferritin to 50–100μg/L), and mobilizable iron/HIC ratio (a marker of total body iron relative to hepatic iron). Exclusion criteria included age less than 16 years or other forms of chronic hepatic disease, including chronic viral hepatitis, immune-mediated hepatic, and known metabolic liver diseases. All individuals were untreated for hemochromatosis at the time of their liver biopsy. Liver biopsy-based fibrosis staging was conducted according to the Scheuer classification by histopathologists with expertise in hemochromatosis: F0-no fibrosis, F1-mild fibrosis with enlarged portal tracts, F2-moderate periportal and portal-portal septa but intact architecture, F3-severe fibrosis with architectural distortion; and F4-cirrhosis with architectural distortion.^18^ For this study, subjects with hepatic fibrosis stages F0-2 were combined and termed ‘low grade fibrosis’, while those with stages F3-4 were combined and termed ‘advanced fibrosis’.

### Hepcidin Measurement

To examine a potential role for hepcidin in the regulation of distribution of iron between hepatic and mobilizable iron stores, we were able to retrieve archival frozen serum samples stored at −80 °C and matched to the date of the original liver biopsy from a small subgroup of 28 of 204 subjects (22 low-grade fibrosis and 6 advanced fibrosis). Serum hepcidin levels were measured using the Intrinsic Hepcidin IDx ELISA kit (Intrinsic Lifesciences), which is a monoclonal antibody-based assay that binds with high affinity to the N-terminus of hepcidin-25. We calculated the serum hepcidin/mobilizable iron and serum hepcidin/HIC ratios as markers of the hepcidin response to mobilizable iron stores and HIC.

### Hepatic Magnetic Resonance Imaging Volumetric Assessment

In order to determine to what extent any variations in mobilizable Fe stores might have been due to variations in liver volume, we undertook an additional study in a separate cohort of 19 newly diagnosed individuals with hemochromatosis across a range of HICs ranging from 59 to 395 (median 109) ***μ***mol Fe/g. Magnetic resonance imaging (MRI) was used to image the liver with a series of axial slices 5 mm thick. The cross-sectional area of the liver was measured by manually segmenting the liver image in each slice. The liver volume was calculated by summing the liver slice volumes (area × 5 mm). The mean HIC for each patient was measured using FerriScan.^19^

The study was approved by the Human Research Ethics Committees of the Royal Brisbane and Women’s Hospital and the QIMR Berghofer Medical Research Institute, Brisbane, Australia; the University of Western Australia; and St John of God Hospital, Perth, Australia, and informed written consent was obtained at the time of entry into the study.

### Statistics

All results are presented as mean ± standard error unless otherwise stated. Categorical data were analyzed using Fisher’s exact test. Continuous data were analyzed using unpaired t-test. Distributions of continuous data were compared with the Kolmogorov-Smirnov test. Simple linear and multiple linear regression models of mobilizable iron as a function of HIC and as a function of the presence of advanced hepatic fibrosis and HIC, respectively, were fitted to the data. The quality of the models was compared using the corrected Akaike information criterion (AIC) in order to take into account the risk of overfitting or underfitting the data when comparing models.^20^ All statistical tests were performed using GraphPad Prism 10.0.2 (GraphPad Software). A *P* value < .05 was considered statistically significant.

## Results

The clinical and biochemical characteristics of the study subjects are shown in Table 1. Of the 204 individuals, 163 (80%) had low-grade fibrosis, while 41 (20%) had advanced fibrosis. Ninety-three percent of the subjects with advanced fibrosis were male compared with 61% of those with stage F0-2 fibrosis (*P* < .0001). Men were much more likely to have advanced fibrosis than women (odds ratio 8.0 [95% confidence interval [CI] 2.5–25.4]). Those with advanced fibrosis were significantly older and had significantly higher alcohol consumption, proportions of individuals with arthritis or diabetes mellitus, serum transferrin saturation and ferritin levels, HIC, HII, and mobilizable iron stores (Table 1). Individuals with advanced fibrosis exhibited 60% greater accumulation of mobilizable iron relative to HIC (0.070 ± 0.008 g Fe/[μmol Fe/g]) compared with those subjects who had low-grade fibrosis (0.044 ± 0.002 g Fe/[μmol Fe/g], *P* < .0001). We examined the relationships between age and HIC, mobilizable iron stores, or mobilizable iron/HIC. Less than 3% of the variance was explained by age in any of these analyses, confirming the absence of any significant effect of age on these parameters. In our small hepcidin subgroup study (n = 28), serum hepcidin levels were 40% lower in those with advanced fibrosis (n = 6) compared with those who had low-grade fibrosis (n = 22), 10.0 ± 1.9 and 16.5 ± 2.1 ng/ml, respectively (*P* = .035). Serum hepcidin/mobilizable iron and serum hepcidin/HIC ratios were 60% and 90% lower in subjects with advanced hepatic fibrosis compared with low-grade fibrosis (Table 1, *P* = .003 and *P* = .01, respectively). Serum bilirubin and albumin levels, used as markers of hepatic synthetic function, were similar in the advanced fibrosis and low-grade fibrosis groups (Table 1).

**Table 1:** Clinical and Biochemical Characteristics of 204 Men and Women With Hemochromatosis With Low Grade vs Advanced Hepatic Fibrosis Stage

Characteristics	Low grade	Advanced	*P*
N	163	41	
Male (N, %)	100,61	38,93	.0001^a^
Age (y)	42 ± 1	50 ± 2	.002
Alcohol (g/d)	21 ± 2	49 ± 7	.0001
Arthritis (N, %)	79,48	40,98	.0001^a^
Diabetes (N, %)	3,2	7,17	.0006^a^
TRS (%)	80 ± 2	89 ± 2	.003
Ferritin (μg/L)	951 ± 56	3088 ± 192	.0001
HIC (μmol Fe/g)	161 ± 8	325 ± 26	.0001
HII ([μmol Fe/g]/y)	4.1 ± 0.2	6.7 ± 0.6	.0001
Mobilizable Fe (g)	6.0 ± 0.3	18.6 ± 1.7	.0001
Mobilizable Fe/HIC (g Fe/[μmol Fe/g])	0.044 ± 0.002	0.070 ± 0.008	.0001
Archival serum substudy			
N	22	6	
Serum hepcidin (ng/ml)	16.5 ± 2.1	10.0 ± 1.9	.035
Serum hepcidin/mobilizable Fe ([ng/ml]/g Fe)	4.6 ± 0.9	1.0 ± 0.5	.003
Serum hepcidin/HIC ([ng/ml]/[μmol Fe/g])	0.13 ± 0.02	0.05 ± 0.02	.01
Bilirubin (μmol/L)	12.4 ± 1.1	16.1 ± 1.8	NS
Albumin (g/L)	40.2 ± 2.2	42.4 ± 0.8	NS

HIC, hepatic iron concentration; HII, hepatic iron index; NS, not significant; TRS, transferrin saturation. For the hepcidin measurements, there were 22 low-grade and 6 advanced hepatic fibrosis subjects with matched serum at the time of liver biopsy suitable for measurement. *P* values are derived from unpaired t test with Welch’s correction unless otherwise specified.

a Fisher’s exact test.

Since almost all subjects with advanced fibrosis were men, we undertook a discrete subgroup analysis of the male group alone (Table 2). Thirty-eight of 138 men (28%) had advanced fibrosis. Overall results were similar to the results observed for men and women combined (Table 2).

**Table 2:** Clinical and Biochemical Characteristics of 138 Men With Hemochromatosis With Low Grade vs Advanced Hepatic Fibrosis Stage

Characteristics	Low grade	Advanced	*P*
N	100	38	
Age (y)	40 ± 1	50 ± 2	.0001
Alcohol (g/d)	28 ± 3	52 ± 8	.0001
Arthritis (N, %)	56,56	37,97	.0001^a^
Diabetes (N, %)	1,1	7,18	.0005^a^
TRS (%)	81 ± 2	90 ± 2	.02
Ferritin (μg/L)	1181 ± 74	3142 ± 203	.0001
HIC (μmol Fe/g)	172 ± 11	318 ± 27	.0001
HII ([μmol Fe/g]/y)	4.5 ± 0.3	6.5 ± 0.7	.002
Mobilizable Fe (g)	7.2 ± 0.4	17.8 ± 1.6	.0001
Mobilizable Fe/HIC (g Fe/[μmol Fe/g])	0.051 ± 0.004	0.067 ± 0.006	.025
Archival serum substudy			
N	6	6	
Serum hepcidin (ng/ml)	20.7 ± 3.8	10.0 ± 1.9	.034
Serum hepcidin/mobilizable FE ([ng/ML]/g Fe)	6.1 ± 2.4	1.0 ± 0.5	.08
Serum hepcidin/HIC ([ng/ml]/[μmol Fe/g])	0.18 ± 0.05	0.05 ± 0.02	.04
Bilirubin (μmol/L)	13.3 ± 1.3	16.6 ± 1.9	NS
Albumin (g/L)	41.2 ± 2.6	42.3 ± 0.9	NS

HIC, hepatic iron concentration; HII, hepatic iron index; NS, not significant; TRS, transferrin saturation. For the male subgroup, there were 7 low-grade and 6 advanced hepatic fibrosis subjects with matched serum at the time of liver biopsy suitable for measurement. *P* values are derived from unpaired t test with Welch’s correction unless otherwise specified.

a Fisher’s exact test.

**Figure 1:**
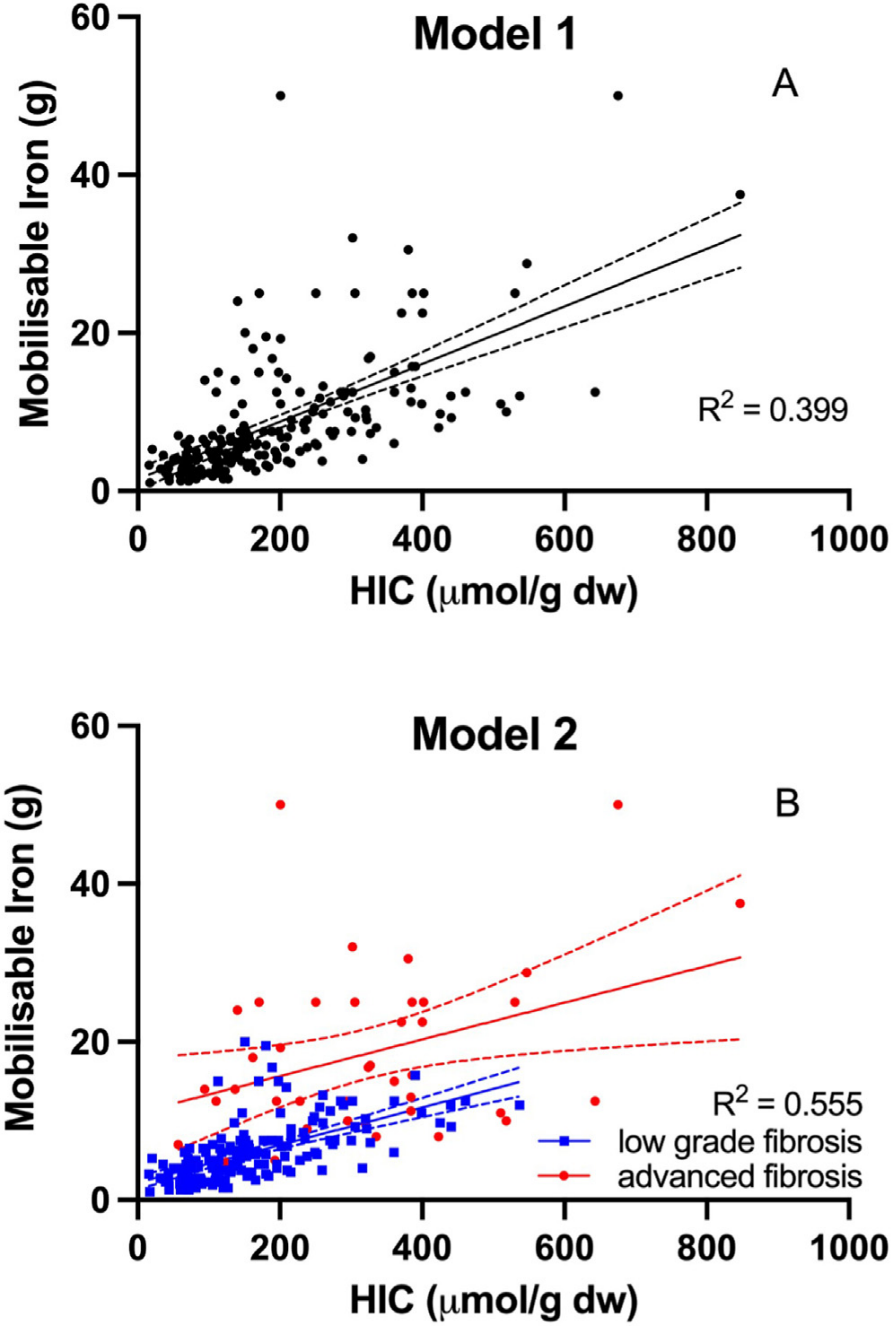
Mobilizable iron plotted against HIC at diagnosis for the 204 hemochromatosis subjects. (A) Data are modeled (model 1) with simple linear regression (solid line) with dashed lines indicating the 95% confidence intervals on the model. (B) Data are modeled (model 2) with multiple linear regression taking into account the presence or absence of advanced hepatic fibrosis as a factor.

A linear model (Model 1) fitted to the mobilizable iron (MobFe) data as a function of HIC for the 204 individuals with hemochromatosis is shown in Figure 1A. The mathematical form of the model is:

MobFe = 1.483 + 0.03645∗HIC (Model 1), with MobFe in units of g Fe and HIC in units of μmol Fe/g. There is a large degree of scatter of the data about the fitted model, with the model explaining only 39.9% of the variance in mobilizable iron.

A more complex multiple linear regression model including the presence of advanced fibrosis as a factor (Model 2) was then fitted to the data (Figure 1B). The mathematical form of the fitted model is:

MobFe = 2.209 + 8.824 ∗F + 0.02373∗HIC – 0.0005237∗F∗HIC (Model 2), where F = 1 when advanced fibrosis is present and F = 0 if advanced fibrosis is not present. The model explains 55.5% of the variance of mobilizable iron and suggests the presence of a discrete phenotype characterized by advanced hepatic fibrosis and enhanced mobilizable iron stores relative to HIC.

In order to determine whether the more complex Model 2 was a better-quality model than Model 1, considering the risk of overfitting the data, the models were compared using the corrected AIC. The difference in the corrected Akaike information criteria was 56.96 indicating that Model 2 is ≥ 10^12^ times as probable to be of better quality than Model 1.

Since an association between advanced fibrosis and age was apparent (Table 1), a multiple linear regression model with age and HIC as factors for predicting mobilizable iron stores (Model 3) was compared with Model 2. Model 3 explained 40.1% of the variance of mobilizable iron, but when compared with Model 2, the corrected AIC difference was 60.5 indicating that Model 2 is ≥ 10^13^ times as probable to be of better quality than Model 3. The distributions of weekly alcohol consumption for subjects with or without advanced hepatic fibrosis had significantly different medians (30 and 10 mg/week, respectively, *P* = .0001, Mann-Whitney test). As such, a multiple linear regression model of mobilizable iron with alcohol consumption and HIC as factors (Model 4) was compared with Model 2 and Model 1. Model 4 was not significantly better than Model 1 and was significantly less likely to be of better quality than Model 2 (with Model 2 being ≥ 10^13^ times more likely to be higher quality). Thus, alcohol consumption is not a significant independent factor determining mobilizable iron in these subjects.

**Figure 2:**
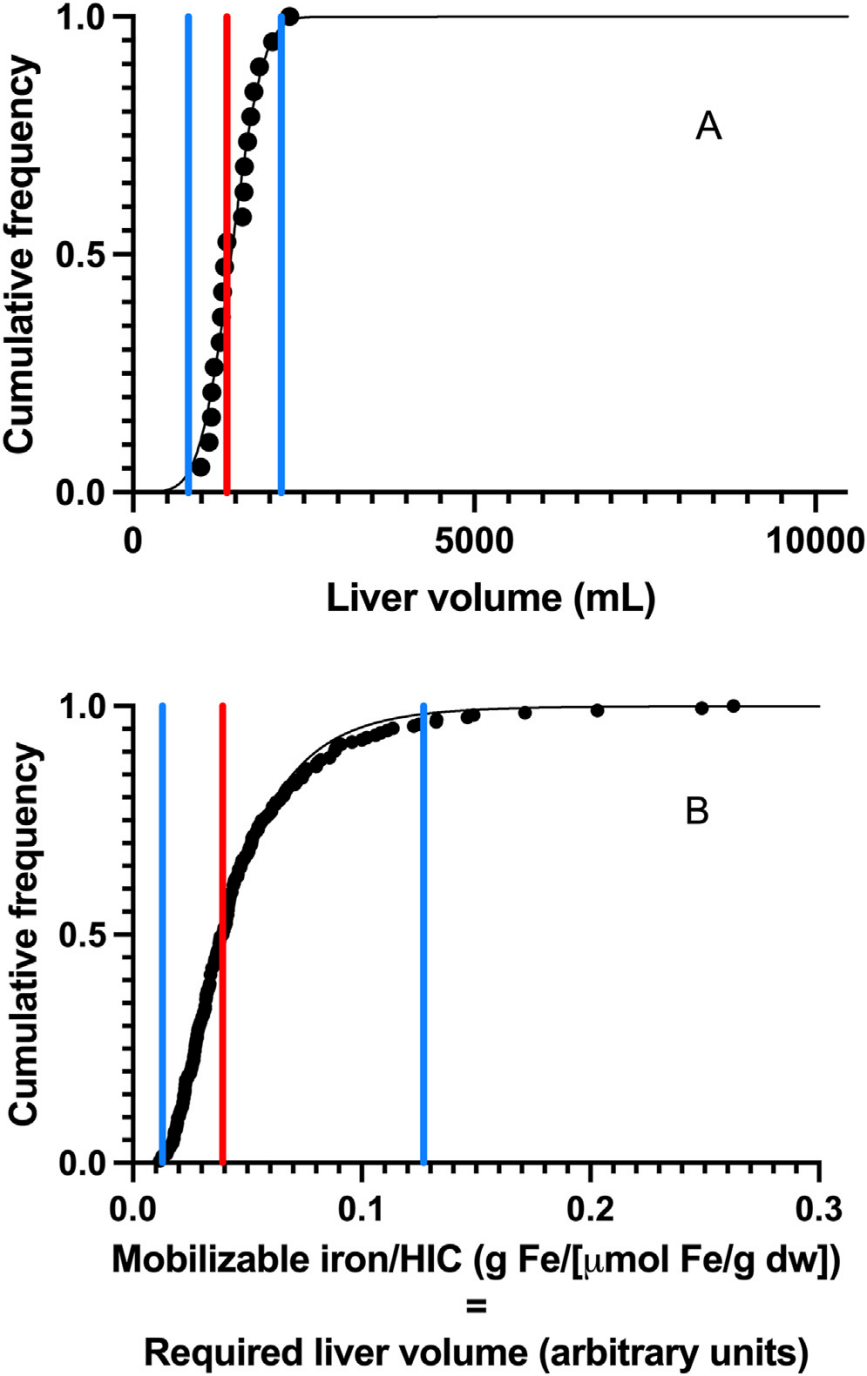
Cumulative distribution of liver volumes measured by MRI for 19 representative hereditary hemochromatosis subjects. (A) The data are modeled with a normal distribution (black solid curve). The median (red line) and the 95% limits of the modeled distribution (blue lines) are shown. (B) Cumulative distribution of mobilizable iron to HIC ratios, which are equivalent to the liver volumes (in arbitrary units) required to explain the variance of the mobilizable iron to HIC ratios for the 204 hereditary hemochromatosis subjects. The data are modeled with a log-normal distribution (black solid curve). The median (red line) and the 95% limits of the modeled distribution (blue lines) are shown. (A) and (B) are scaled so that zero and the median coincide for both plots.

Finally, we evaluated whether the variation in mobilizable iron for a given HIC (ie, variation in mobilizable iron to HIC ratio) could possibly be explained by variation in liver volume between individuals. Larger liver volumes for the same HIC would be expected to result in greater mobilizable iron. In order to assess the likelihood that variation in liver volume could be the sole explanation for the observed variation in mobilizable iron to HIC ratios, the distribution of liver volumes was measured using MRI for a separate but representative group of 19 newly diagnosed hemochromatosis patients (12 men and 7 women) with HIC measured by MRI across the spectrum of iron loading ranging from 59 to 394 ***μ***mol/g (Figure 2A). The distribution of HICs was not significantly different from the main study group (KS statistic 0.25, *P* = .24) and the proportion of men (63%) was not significantly different from the main study group (68%) (*P* = .80). Liver volumes were normally distributed with a mean of 1496 mL and a standard deviation of 347 mL (coefficient of variation = 23%) and range from 994 to 2295 mL (Figure 2A).

The distribution of mobilizable iron to HIC ratios in the main cohort of subjects was log-normally distributed with a geometric mean of 0.0395 g Fe/(μmol Fe/g) and a range from 0.010 to 0.263 g Fe/(μmol Fe/g) (coefficient of variation 74%, Figure 2B). Using the normal distribution model for the liver volumes and the log-normal model for the distribution of mobilizable iron to HIC ratios, the ratio of the upper to lower 95% limits of the distributions were 2.7 (95% CI 2.3–3.0) for the liver volumes and 9.7 (95% CI 8.0–11.7) for the mobilizable iron to HIC ratios indicating that the distribution of liver volumes measured by MRI is not sufficiently wide to explain the variability in mobilizable iron to HIC ratios (Figure 2).

## Discussion

It has long been known that the development of iron overload and clinically significant disease is highly variable in individuals with HFE-hemochromatosis. Iron overload-related disease occurs in up to 13% of women and 40% of men, with up to 8% of women and 25% of men presenting with clinically significant liver disease at diagnosis.^1^^,^^2^ The risk of advanced hepatic fibrosis is greatest in those with serum ferritin levels greater than 1000 μg/L, HIC greater than 200 μmol Fe/g, and total mobilizable iron stores greater than 9.6 g.^7^^,^^12^, ^13^, ^14^^,^^17^^,^^21^ Men, especially those with advanced hepatic fibrosis, are at significantly increased risk of mortality from hepatocellular carcinoma.^1^^,^^6^ Thus, accurate assessment for the presence of advanced hepatic fibrosis is important in the management of hemochromatosis. In our study of 204 individuals with hemochromatosis and well-characterized liver biopsy-staged low-grade or advanced hepatic fibrosis, we showed that subjects who develop advanced hepatic fibrosis accumulate much higher mobilizable iron stores relative to HIC compared with those who develop low-grade fibrosis. Furthermore, our detailed multiple linear regression analyses strongly support the concept of a discrete phenotype characterized by the presence of advanced fibrosis and elevated mobilizable iron stores relative to HIC (Figure 1B). While variation in liver volume between individuals may contribute to the variance of mobilizable iron relative to HIC, the observed variation in liver volume in a comparable cohort of subjects is far narrower than that required to explain the observed variance in mobilizable iron relative to HIC (Figure 2). It is possible that biopsy sampling error could account for some of the observed variance in mobilizable iron relative to HIC. While it is known that hepatic fibrosis increases sampling error on chemical assays of HIC,^22^ the error is approximately equally likely to be positive or negative. We have confirmed this in a study of 233 cases with a range of fibrosis stages by comparing biopsy measures of HIC against MRI measures of HIC, which sample much larger volumes than the biopsy.^23^ Hence, the effect of biopsy sampling errors would be to increase the scatter on the mobilizable iron vs HIC graph (Figure 1) uniformly about the regression line rather than to increase the average mobilizable iron to HIC ratio for advanced hepatic fibrosis. Thus, sampling error on HIC measurements from biopsy does not explain the key observation of the study that patients with advanced fibrosis have significantly higher mobilizable iron levels. We believe the additional mobilizable iron in subjects with advanced hepatic fibrosis is most likely present in extrahepatic locations. A readily accessible extrahepatic site of substantial iron storage that can be mobilized by phlebotomy therapy in HFE-hemochromatosis is the bone marrow. This is plausible based on early studies of hemochromatosis prior to discovery of the underlying genetic mutation, which demonstrated bone marrow iron overload in up to 60% of subjects with significant iron overload and cirrhosis^15^ and a dissociation in the rate of iron accumulation between the bone marrow and liver in clinical hemochromatosis, with excess iron being deposited predominantly and preferentially in the liver until the later stages of disease when bone marrow deposition occurs.^16^ Cardiac deposition has also been reported in iron overload disorders, but as we did not perform cardiac MRI measurements of iron concentration, we are unable to comment specifically on the extent to which this may contribute to extrahepatic iron loading.^1^

Given the known suppression of hepatic^24^ and serum^25^ hepcidin levels in hemochromatosis and the potential antifibrogenic effects of hepcidin,^26^ we measured levels of serum hepcidin in archival serum samples that were obtained at the time of liver biopsy. Unfortunately, we were able to retrieve only 28 samples from 204 individuals (22 low-grade fibrosis and 6 advanced fibrosis). In this limited sub-study, we found that subjects with advanced hepatic fibrosis had significantly lower levels of serum hepcidin, hepcidin/mobilizable iron store, and hepcidin/HIC ratios compared with those who had low-grade fibrosis. What causes such significantly lower serum hepcidin levels despite the development of substantial iron overload remains unclear and ideally should be confirmed in larger studies. While hepcidin production is significantly abrogated in hemochromatosis, it is not completely eliminated,^24^^,^^25^ indicating that compensatory systems independent of HFE *per se* remain functional. It is highly likely that hemochromatosis individuals with high mobilizable iron stores have elevated nontransferrin-bound iron levels,^27^^,^^28^ which have been shown to influence hepcidin production by effects on hepatocytes and endothelial cells.

Our study has strengths and limitations. Strengths include the large cohort of subjects with detailed clinical, biochemical, and pathological information, with liver biopsy-staged fibrosis recorded at diagnosis of hemochromatosis, and a subset of patients with matched serum and liver biopsy data. Limitations include the retrospective nature of the study and the potential impact of restricted numbers of subjects who had archival serum available for the serum hepcidin measurements.

## Conclusion

We show that advanced hepatic fibrosis develops in HFE-hemochromatosis individuals with enhanced total body iron loading relative to HIC, consistent with extrahepatic mobilizable iron stores in the bone marrow. These individuals also have low levels of hepcidin relative to HIC raising the possibility that loss of hepcidin’s negative regulatory actions may contribute to both iron loading and fibrogenesis in hemochromatosis.
